# Takotsubo Syndrome Following Inhalation of K4: A Case Report

**DOI:** 10.7759/cureus.102819

**Published:** 2026-02-02

**Authors:** Leandro M Valente, Gabrielle Santos, Ricardo Velho, José Filipe Santos, Inês Gonçalves, Pedro D Lopes, Mariana Guerra, Lurdes M Correia

**Affiliations:** 1 Internal Medicine Department, Centro Hospitalar e Universitário de Coimbra, Coimbra, PRT

**Keywords:** case report, emergency room, stress cardiomyopathy, synthetic cannabinoids, takotsubo syndrome

## Abstract

Takotsubo syndrome (TS), also known as stress cardiomyopathy, is a clinical condition characterized by transient left ventricular dysfunction in the absence of coronary artery disease findings. The most widely accepted mechanism suggests a trigger, either physical or emotional stress, which causes catecholamine release and results in myocardial stunning and contractility alterations. Clinically and analytically, it can mimic acute coronary syndromes. Synthetic cannabinoids, such as the inhaled drug K4, are more powerful than phytocannabinoids and act as strong agonists of cannabinoid receptors, stimulating the sympathetic nervous system, which can serve as a trigger for TS. We report a case of a 59-year-old man with a personal history of schizophrenia and drug addiction, admitted to the emergency room for severe dyspnea and psychomotor agitation. Prior to the onset of symptoms, he had reportedly used the inhaled drug K4. Due to markedly elevated high-sensitivity troponin I and electrocardiographic changes suggestive of myocardial ischemia, an echocardiogram was performed, revealing severe left ventricular dysfunction with mid-apical akinesia of all cardiac walls. Cardiac catheterization showed no significant coronary disease. The patient was diagnosed with TS. After excluding other potential triggers, the use of inhaled K4 was considered the most likely trigger for TS.

## Introduction

Takotsubo syndrome (TS), also known as stress cardiomyopathy, Gebrochenes-Herz syndrome, or broken-heart syndrome, is a form of non-ischemic cardiomyopathy characterized by acute heart failure with typically reversible left ventricular dysfunction [1,2]. The main causes of TS include intense stress, whether psychological or physical, which leads to sympathetic nervous system stimulation and elevated catecholamine release, inducing myocardial stunning [3,4].

Synthetic cannabinoids, much like phytocannabinoids, act as agonists of the cannabinoid receptors (CB1 and CB2), stimulating the sympathetic nervous system and leading to increased catecholamine levels [5,6]. However, synthetic cannabinoids used as drugs of abuse, such as K4 - a member of the newest generation of synthetic agonists - exhibit a binding affinity and potency significantly higher than those of naturally occurring phytocannabinoids like delta-9-tetrahydrocannabinol (delta-9 THC). Consequently, this increased pharmacological potency is directly associated with a heightened risk of severe morbidity and mortality compared to natural cannabis use [7,8].

The drug K4 is part of a group of synthetic cannabinoids that have emerged in recent years. Although the cannabinoid concentration in synthetic products can vary widely, these compounds can have a potency up to 100 times greater than that of phytocannabinoids [7]. Although TS has been described as a possible consequence of cannabinoid use, the potency of newer synthetic cannabinoids makes them extremely dangerous [5,7]. We report the case of a 59-year-old man who developed stress-induced cardiomyopathy after inhaling a synthetic cannabinoid.

## Case presentation

A 59-year-old man with a medical history of schizophrenia, severe hearing loss, and drug addiction, without known allergies, on chronic medication with clozapine (100 mg once daily) and amisulpiride (50 mg once daily), presented to the emergency department with severe dyspnea. Upon admission to the emergency room, the patient was diaphoretic, tachypneic, and exhibited accessory muscle use (intercostal retraction and abdominal breathing). Clinical signs included a blood pressure of 117/71 mmHg (mean blood pressure 86 mmHg), heart rate of 110 beats per minute (bpm), and a peripheral oxygen saturation of 70% while on a 28% Venturi mask. Arterial blood gas analysis confirmed severe hypoxemia with a PaO_2_/FiO_2_ ratio of 178 mmHg, alongside metabolic acidosis (pH 7.263, HCO_3_ 15.9 mEq/L, and lactate 4.34 mmol/L). According to the Berlin criteria, the patient’s clinical presentation and oxygenation index were consistent with moderate acute respiratory distress syndrome (ARDS). However, the possibility of acute cardiogenic pulmonary edema was also considered, particularly in the context of a potential TS secondary to substance use. To investigate potential infectious triggers, two sets of blood cultures and a urine culture were collected upon admission, all of which subsequently yielded negative results.

**Figure 1:**
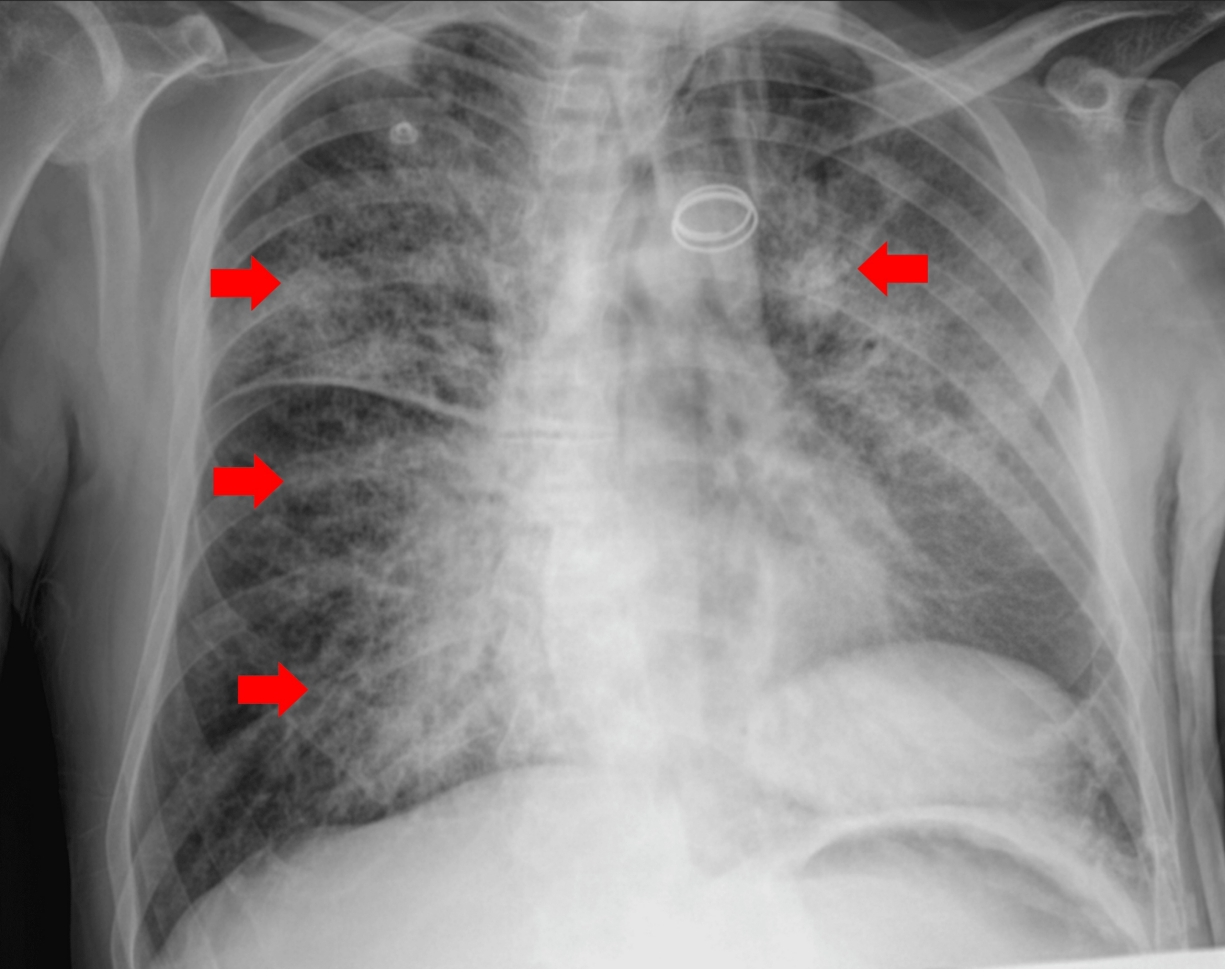
Chest X-ray showing a diffuse bilateral reticulonodular pattern (red arrows), consistent with the patient’s presentation of acute respiratory distress syndrome (ARDS).

Due to impending respiratory failure from ARDS, non-invasive mechanical ventilation (NIMV) was initiated. A trial of NIMV was attempted initially to manage the severe hypoxemia and the potential cardiogenic component of the pulmonary edema, while avoiding immediate sedation in a patient with psychomotor agitation. The NIMV was started in spontaneous/timed (S/T) mode with an inspiratory positive airway pressure (IPAP) of 16, expiratory positive airway pressure (EPAP) of 6, and FiO_2_ of 100%. The chest X-ray showed a diffuse bilateral reticulonodular pattern (Figure 1), consistent with the patient's ARDS presentation.

The patient admitted to having consumed the K4 drug by inhalation hours before the onset of symptoms. However, the exact timing and potential concomitant use of other substances could not be determined. Furthermore, his rapid and progressive cognitive decline upon admission precluded a detailed medical history, leaving his long-term drug use and previous exposure to K4 unknown.

The Poison Information Center (CIAV) was contacted, and information about the synthetic cannabinoid and its potential complications, particularly cardiac ones, was provided.

Laboratory analysis showed a leukocytosis of 24.8 × 10^9^/L with neutrophilia of 21.56 × 10^9^/L, a high-sensitivity troponin I (hsTnI) of 2695.0 ng/L, and N-terminal pro-brain natriuretic peptide (NT-proBNP) of 13732 pg/mL, with no other significant changes (Tables 1-2).

**Table 1:** Patient's complete blood count on admission. WBC: white blood cell count

Complete blood count		
Parameter	Value	Reference
WBC	24.80 x 10^9/L	3.90-10.20 x 10^9/L
Neutrophils	21.56 x 10^9/L	1.50-7.70 x 10^9/L
Lymphocytes	1.97 x 10^9/L	1.10-4.50 x 10^9/L
Hemoglobin	15.4 g/dL	12.0-15.6 g/dL
Platelets	546 x 10^9/L	150-450 x 10^9/L

**Table 2:** Patient's serum chemistry on admission. BUN: blood urea nitrogen; CRP: C-reactive protein; hsTnI: high-sensitivity troponin I; NT-proBNP: N-terminal pro-brain natriuretic peptide

Serum chemistry		
Parameter	Value	Reference
Creatinine	0.90 mg/dL	0.72-1.18 mg/dL
BUN	12.6 mg/dL	7.9-20.9 mg/dL
Sodium	133 mmol/L	136-146 mmol/L
Potassium	3.0 mmol/L	3.5-5.1 mmol/L
CRP	6.32 mg/dL	<0.50 mg/dL
hsTnI	2695.0 ng/L	<1.9 ng/L
NT-proBNP	13732 pg/mL	<125 pg/mL

**Figure 2:**
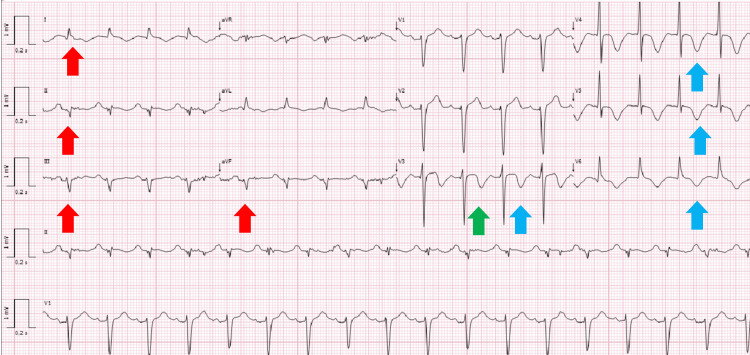
Electrocardiogram demonstrating a left anterior fascicular block (red arrows), T-wave inversion in the precordial leads (blue arrows), and a prolonged QTc interval (green arrow).

The electrocardiogram revealed a left anterior fascicular block, T-wave inversion in the precordial leads, and a prolonged QTc interval (Figure 2).

Echocardiogram revealed severe left ventricular dysfunction with mid-apical akinesia of all walls and basal hypercontractility. Considering the severity of the clinical picture, the echocardiogram was performed at the patient's bedside in the emergency room, so no images were recorded. No baseline echocardiograms were available, as the patient had no prior cardiovascular history.

While in the emergency department, the patient's respiratory failure worsened, requiring invasive mechanical ventilation. He also developed marked hypotension in the context of cardiogenic shock, which required vasopressor support with norepinephrine.

**Figure 3:**
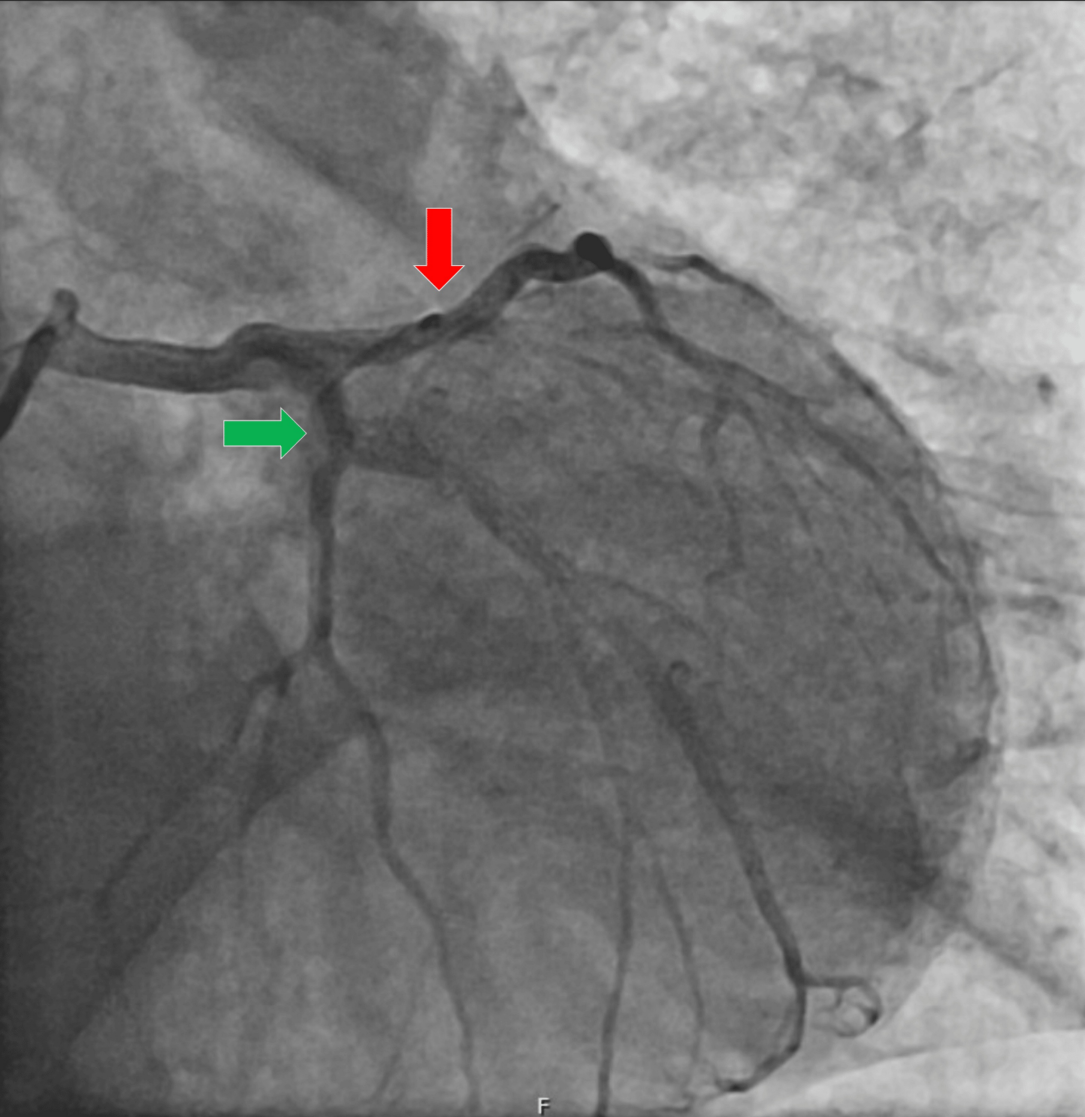
Cardiac catheterization findings. The left main coronary artery had no lesions. The left anterior descending artery (red arrow) showed irregularities but no significant lesions. The circumflex artery (green arrow) had irregularities but no significant lesions. The right coronary artery (not shown) was dominant and showed no lesions. Ventriculography revealed a non-dilated left ventricle with akinesia of the mid-apical segments and basal hypercontractility, suggestive of Takotsubo syndrome. There was no mitral insufficiency.

Cardiac catheterization showed no evidence of coronary artery disease (Figure 3).

The patient was admitted to the Coronary Intensive Care Unit (CICU) for targeted treatment and further investigation. However, on the second day of hospitalization, despite transient clinical improvement, he experienced a cardiac arrest with asystole. Advanced life support was initiated, but the clinical situation could not be reversed, and he ultimately passed away.

## Discussion

Although the pathophysiology of TS is not fully understood, the most widely accepted mechanism involves a trigger, either psychological or organic, that causes a surge in circulating catecholamines and their metabolites, leading to transient systolic and diastolic left ventricular dysfunction [3,4]. This is typically accompanied by elevated cardiac injury enzymes and electrocardiographic changes suggestive of myocardial ischemia [1,9]. This syndrome often presents clinically and biochemically similar to acute coronary syndrome, with elevated cardiac enzymes and electrocardiographic ischemic changes, but without structural abnormalities detected on coronary angiography [10,1]. Although usually reversible, TS can lead to severe complications, including cardiogenic shock, left heart failure, ventricular arrhythmias, and ventricular wall rupture. It carries an associated mortality rate of 3-4% [2]. Various known triggers have been described, including emotional stress, such as intense psychological events, and organic triggers, such as extensive burns, head trauma, surgery, and stimulant drug use [1,2].

TS is classified into four main types based on the pattern of myocardial wall motion abnormalities. The typical form, characterized by apical ballooning, is the most prevalent. Atypical variants include the midventricular type, the basal (or reverse) type, and the rare focal type, which involves isolated segments of the left ventricle. In this case, the echocardiogram revealed severe left ventricular dysfunction with mid-apical akinesia, consistent with the typical apical ballooning presentation.

Synthetic cannabinoids, such as the inhaled drug K4, act as potent agonists of cannabinoid receptors, stimulating the sympathetic nervous system. Cardiovascular effects include tachycardia, resulting from increased cardiac chronotropy, and decreased oxygen supply due to high levels of circulating carboxyhemoglobin. This creates a mismatch between oxygen demand and supply at the coronary level, leading to transient myocardial ischemia.

The drug's potent agonistic effect on cannabinoid receptors, particularly CB1, triggers a massive surge of catecholamines, which is the primary cause of TS [3,4]. This hyperadrenergic state leads to transitory ventricular dysfunction, especially on the left ventricle's apex, causing it to balloon out while the base remains contracted. Regarding management, although the pathophysiology is catecholamine-driven, the use of norepinephrine as a first-line vasopressor was justified by the need to maintain adequate mean arterial pressure and cerebral perfusion in the setting of refractory shock, despite the theoretical risk of exacerbating the hyperadrenergic state.

Limitations

This case report has inherent limitations. First, due to the patient's rapid and progressive cognitive decline and psychomotor agitation upon admission, a detailed and reliable medical history regarding long-term drug use or previous exposure to K4 could not be fully obtained. Second, the lack of a formal, comprehensive toxicology screening meant that the presence of other concomitant substances could not be definitively excluded. Finally, since the bedside echocardiogram was performed under emergency conditions to guide immediate life-saving interventions, no video loops or images were recorded for retrospective review. Despite these constraints, the strong temporal correlation between the reported inhalation of K4 and the onset of symptoms suggests it was the primary trigger for the syndrome.

Nevertheless, the link between K4 and TS highlights the dangerous cardiovascular risks associated with these unregulated and highly potent synthetic drugs [5,6].

## Conclusions

This case report details a patient who developed TS after inhaling the synthetic cannabinoid K4. While TS often has a favorable outcome and most patients recover fully, this case demonstrates the potential for severe complications, including cardiogenic shock and death. To our knowledge, this is the first reported case of TS triggered specifically by the synthetic cannabinoid K4, emphasizing the extreme potency of these novel substances and the need for increased awareness among healthcare professionals regarding their serious and potentially fatal cardiovascular effects.
